# Impact of intrapartum antibiotic prophylaxis on the oral and fecal bacteriomes of children in the first week of life

**DOI:** 10.1038/s41598-024-68953-z

**Published:** 2024-08-06

**Authors:** Eliska Pivrncova, Lucie Buresova, Iva Kotaskova, Petra Videnska, Lenka Andryskova, Pavel Piler, Petr Janku, Ivo Borek, Jan Bohm, Jana Klanova, Eva Budinska, Petra Borilova Linhartova

**Affiliations:** 1 RECETOX, Faculty of Science, Masaryk University, Kotlarska 2, Brno, Czech Republic; 2 BioVendor MDx, Karasek 1, Brno, Czech Republic; 3 Department of Gynecology and Obstetrics, University Hospital Brno, Jihlavska 20, Brno, Czech Republic; 4 Department of Gynecology and Obstetrics, Faculty of Medicine, Masaryk University, Kamenice 5, Brno, Czech Republic; 5 Department of Neonatology, University Hospital Brno, Jihlavska 20, Brno, Czech Republic; 6 Department of Neonatology, Faculty of Medicine, Masaryk University, Kamenice 5, Brno, Czech Republic; 7 Clinic of Maxillofacial Surgery, University Hospital Brno, Jihlavska 20, Brno, Czech Republic

**Keywords:** Microbiome, Infant, Mother, Next-generation sequencing, Antibiotics, 16S rRNA, Diversity, Microbiology, Molecular biology

## Abstract

Intrapartum antibiotic prophylaxis (IAP) is commonly used during C-section delivery and in Group B *Streptococcus*-positive women before vaginal delivery. Here, we primarily aimed to investigate the effect of IAP on the neonatal oral and fecal bacteriomes in the first week of life. In this preliminary study, maternal and neonatal oral swabs and neonatal fecal (meconium and transitional stool) swabs were selected from a pool of samples from healthy mother-neonate pairs participating in the pilot phase of CELSPAC: TNG during their hospital stay. The DNA was extracted and bacteriome profiles were determined by 16S rRNA amplicon sequencing (Illumina). In the final dataset, 33 mother-neonate pairs were exposed to antibiotics during C-section or vaginal delivery (cases; +IAP) and the vaginal delivery without IAP (controls, -IAP) took place in 33 mother-neonate pairs. Differences in alpha diversity (Shannon index, p=0.01) and bacterial composition (PERMANOVA, p<0.05) between the +IAP and -IAP groups were detected only in neonatal oral samples collected ≤48 h after birth. No significant differences between meconium bacteriomes of the +IAP and -IAP groups were observed (p>0.05). However, the IAP was associated with decreased alpha diversity (number of amplicon sequence variants, p<0.001), decreased relative abundances of the genera *Bacteroides* and *Bifidobacterium*, and increased relative abundances of genera *Enterococcus* and *Rothia* (q<0.01 for all of them) in transitional stool samples. The findings of this study suggest that exposure to IAP may significantly influence the early development of the neonatal oral and gut microbiomes. IAP affected the neonatal oral bacteriome in the first two days after birth as well as the neonatal fecal bacteriome in transitional stool samples. In addition, it highlights the necessity for further investigation into the potential long-term health impacts on children.

## Introduction

Birth events and factors unique to the labor may influence the composition of the neonate’s microbiome. The mode of delivery and use of antibiotics count among the factors most affecting neonate colonization by microorganisms^1–4^. In the last decade, the research in gut microbiome acquisition has been consistently reporting that C-section (cesarean section, CS) decreases the relative abundance of *Bacteroides* and *Bifidobacterium* spp. and increases the abundance of *Enterococcus*, *Staphylococcus*, and/or *Clostridioides* spp. in the gut microbiome of CS-delivered neonates^5–7^. A similar pattern was observed in the neonatal oral microbiome in children delivered by CS, which mirrors communities similar to those found on the mother’s skin^8^. Vaginal mode of delivery (VD) is considered an important primary source of microbial communities for the children´s long-term health because it promotes a more diverse and beneficial microbiota than CS^1,9–11^. However, microbial profile differs even among neonates after vaginal births. One of the causes for this may lie in intrapartum antibiotics prophylaxis (IAP), the most frequent cause of exposure to antibiotics during the perinatal period and labor^12^.

The IAP treatment is an important intervention to reduce the risk of maternal and neonatal postnatal infection. It is commonly used in CSs but also important in VDs to prevent the transmission of any pathogens in suspected infection, mostly due to maternal group B *Streptococcus* (GBS) positivity or, in some cases, due to premature rupture of membranes^13^. The incidence of CSs globally increases, as well as the IAP use. According to research by the World Health Organization^14^, the global CS rate increased from approx. 7 % in 1990 to 21 % in 2021. The estimated worldwide prevalence of GBS during pregnancy is 18 %, despite regional variability ranging from 11 to 35%^15^. Even though the screening procedures for GBS contributed to reducing the incidence of early-onset GBS sepsis in neonates^16,17^, it also increased the use of IAP^18^. In 2002, the Center for Disease Control and Prevention (CDC) recommended a universal screening-based strategy in their guidelines, where all pregnant women are screened for GBS between 36 and 38 weeks of pregnancy^17,19^. In effect, an increase of IAP from 26.8 % in 1998–1999 to 31.7 % in 2003–2004 was reported in the USA^20^. In contrast, some countries (Denmark, UK, New Zealand) employed GBS-mitigating strategies based on the presence of risk factors for early-onset GBS disease in neonates, while not clearly lowering the antibiotic administration rates^19^. IAP, however, changes the microbiome acquisition trajectory. It may directly affect microbial colonization by passing antibiotics into the fetal/neonatal bloodstream through placenta or through breast milk. Moreover, it reduces the transmission of susceptible bacterial groups from the mother to the neonate^10,21,22^.

The gut microbiome has been shown to affect childhood development. Antibiotic-mediated gut dysbiosis in neonates is associated with increased health risks, such as allergy^23,24^ and obesity^25^. Despite this knowledge, the effects of IAP on neonatal oral microbiome in the first week of life remains underexplored^26^ and of the available studies, most focused on the changes of the neonatal fecal bacteriome only two days or later after birth^11,12,21,27–30^. To this date, only two studies describing the impact of IAP on the bacteriome of meconium samples have been published^27,31^. The presented preliminary study aimed to investigate the effect of IAP on the bacteriome profiles of the (i) maternal oral mucosa, (ii) neonatal oral cavity, as well as the neonatal (iii) meconium and (iv) transitional stool in the first week after birth.

## Methods

### Study population

The Central European Longitudinal Studies of Parents and Children: The Next Generation (CELSPAC: TNG) study is designed as a new prospective birth cohort which will follow up on 2,000 children from their prenatal period to adolescence with the aim of assessing exposome factors affecting children’s health^32^. A pilot phase of CELSPAC: TNG study was initiated to evaluate feasibility of the protocol for collection, processing and storing of biological samples including oralswabs. The mothers (age ≥18 years) were recruited in their 38th week of pregnancy at University Hospital Brno, Czech Republic, in 2015 and 2016. In line with Helsinki declaration, all pregnant women involved in that study were willing to participate and gave informed consent for themselves and prospective neonates. Data related to the pregnancy, maternal IAP, birth, and mother’s and neonate’s health characteristics were retrieved from hospital records.

**Figure 1:**
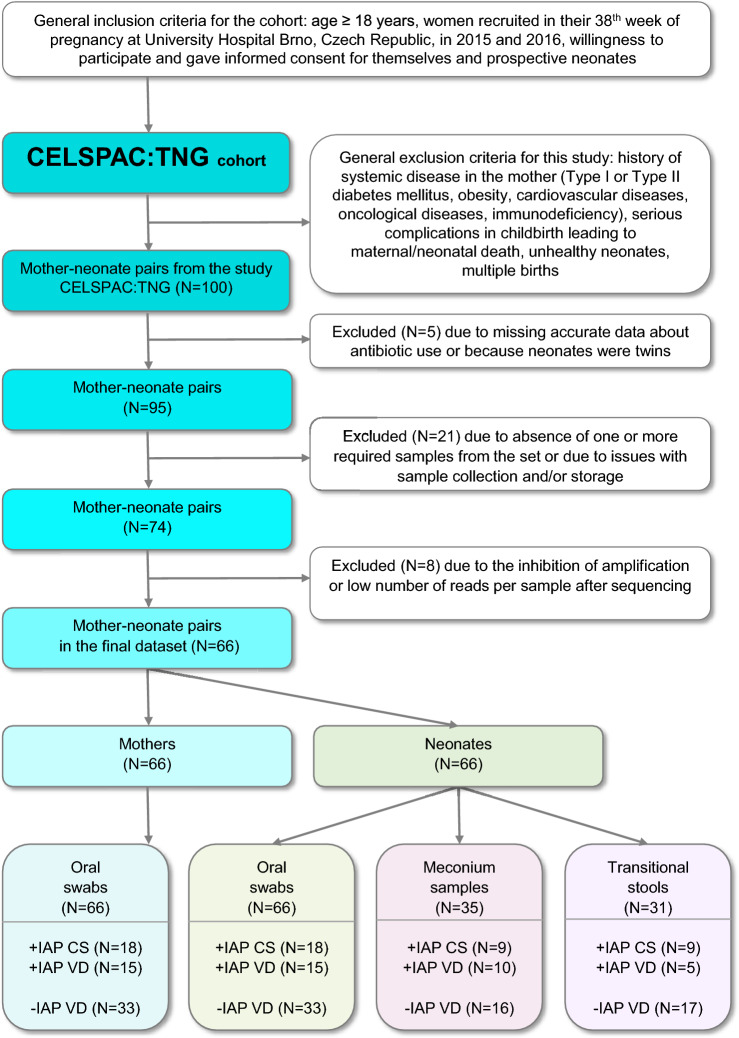
Inclusion and exclusion criteria flowchart and overview of analyzed samples. *CELSPAC: TNG* Central European Longitudinal Studies of Parents and Children: The Next Generation Study, *N* number of participants.

From that cohort, we retrospectively selected 100 mother-neonate pairs. For mothers, exclusion criteria comprised a history of systemic disease (Type I or Type II diabetes mellitus, obesity, cardiovascular diseases, oncological diseases, immunodeficiency; gestational diabetes and asthma were, however, not considered exclusion criteria) and serious complications in childbirth leading to maternal/neonatal death. For neonates, the inclusion criteria were: birth in gestational weeks 38–42 by CS or VD and good health without congenital defects. Multiple births were not included in the study. From thus acquired cohort of 100 mother-neonate pairs, 26 pairs were removed based on additional exclusion criteria, i.e., the absence of crucial data (IAP type and dose, details about sample collection, and sample storing) or the absence of one or more samples from the pair. Lastly, additional eight mother-neonate pairs were removed from the cohort due to the lack of sequencing depth analysis in one or more samples from the mother-neonate pair, yielding a final cohort of 66 pairs, see Fig. 1.

The current preliminary study, focusing on the transmission and development of bacterial community in relation to exposure factors in children in the first week of their life, was designed as a retrospective case-control association study. Mother-neonate pairs were retrospectively classified into 2 groups according to the IAP administration, i.e., cases with IAP (+IAP), and controls without IAP (-IAP).

### Sample collection

Healthcare professionals collected all specimens in a sterile and uniform manner within the first 5 days of postpartum hospitalization. Swabs from the oral cavity of each mother (buccal mucosa) and neonate were collected into sterile 1.5 mL tubes by the nylon swab FLOQSwabs (Copan, CA, USA) and stored at −80 ℃ until DNA extraction.

The swabs from the single-use diaper were collected by FLOQSwabs (Copan, CA, USA) and classified as meconium or transitional stool by experienced healthcare professionals based on fecal characteristics (color and consistency). Therefore, hereinafter, we strictly distinguish between the expressions “meconium” and “transitional stool” describing a non-meconium sample.

### DNA extraction

All samples were processed using the DNeasy® PowerSoil® kit (Qiagen, Germany), which had been proven suitable for clinical sample extraction before^33–35^. 750 uL of bead solution and 60 uL of C1 buffer heated to 64 ℃ were added to the broken swabbing head. The FastPrep-24 (MP Biomedicals, USA) was used for sample homogenization and set to 6.5 m/s for 45 s. The next steps were performed according to the manufacturer’s manual. The genomic DNA concentration was determined spectrophotometrically using Synergy Mx (BioTek, USA). The quality of purified genomic DNA was determined after electrophoresis on 1% agarose gel. Extracted DNA was stored at −20 ℃.

### 16S rRNA amplicon sequencing

Substantial improvement was made to the library preparation process, when the polymerase chain reaction (PCR) reagents´ decontamination step using the 8-methoxypsoralen (8-MOP) was applied, as used earlier for next-generation sequencing (NGS) analysis of low-abundant samples^36^. Prepared PCR mixtures with 8-MOP (0.16 mM, Sigma-Aldrich, USA) were incubated at 4 ℃ for 1.5 h and exposed to UVA (365 nm) for 7 min (30 J/cm^2^) in UV-crosslinker. After decontamination, the template and the artificial spike-in standard (SIS) were added to each PCR. The spike-in standard (SIS)^37^ was used as the internal control and consisted of the synthetic *16S rDNA* gene (1525 bp). The synthetic gene was inserted into the pUC57-Amp vector (GenWiz, Germany), transformed into Dh5α *Escherichia coli* cells, and cloned. The plasmids with artificial sequence were extracted and the exact copy numbers per 1 μL were assessed. Each PCR was spiked with 200 copies of SIS. The sequencing library was prepared according to the Illumina 16S Metagenomic Sequencing Library Preparation Protocol with some deviations regarding the widely used double barcoding strategy, as described before^33^. The V3-V4 hypervariable region of the bacterial *16S rDNA* gene approx. 290 bp long was amplified using the previously published degenerated primers^38^ with the inner tags for distinguishing the particular samples. The total volume of 31 µL consisted of 15 uL of Q5® High Fidelity Master Mix (New England Biolabs, MA, USA), 1.5 µL of each primer (10 uM), 2 µL of 8-MOP 10x diluted, 1 µL of SIS and 5 µL of extracted DNA and 5 µL of sterile DNA-free water (Qiagen, Germany). The initial denaturation (15 min at 95 ℃) was followed by 30 cycles consisting of denaturation at 94 ℃ for 35 s, primer annealing at 55 ℃ for 35 s, and extension at 72 ℃ for 45 s. The final extension at 72 ℃ lasted 10 min. The PCR negative (sterile DNA-free water used as a template) and in-house positive controls (mixture of stool samples with high load of bacterial DNA) were included in each PCR batch. PCR products were visualized after electrophoresis on 1.5 % agarose gel.

SPRIselect beads (Beckman Coulter Genomics, USA) were used to clean the PCR products according to the manufacturer’s recommendations. Quant-iT (Thermo Fisher Scientific, USA) and microplate reader Synergy Mx (BioTek, USA) were used to fluorochemically assess the concentration of cleaned PCR products to pool those with different inner tags equimolarly. Pools were indexed with Nextera XT indexes (Illumina, USA), quantified fluorochemically, and pooled equimolarly. The quality and concentration of the prepared library was assessed using a 2100 Bioanalyzer Instrument (Agilent Technologies, USA) and qPCR (KAPA Library Quantification Complete Kit, Kapa Biosystems, USA) shortly before sequencing. The library was diluted to a final concentration of 8 pM, and 20% of PhiX DNA (Illumina, USA) was added. Sequencing was performed with the Miseq reagent kit V3 using a MiSeq 2000 instrument according to the manufacturer’s instructions (Illumina, USA).

### Bioinformatics processing

Paired reads from 16S rRNA amplicon sequencing were first processed using an in-house pipeline implemented in Python 3. Our in-house script used Trimmomatic to trim bases with a quality score below Q20, to remove adapters and barcodes, while maintaining only paired-end reads with minimum overlap of 20 with the trimming settings set to—forward trimmed to 236 bp and—reverse trimmed to 168 bp. In order to minimize sequencing and PCR-derived errors, forward and reverse reads were denoised using the DADA2 amplicon denoising R package^39^. Following denoising, the forward and reverse reads were joined using the fastq-join read joining utility. To be joined, reads in pairs had to overlap in at least 20 base pairs with no mismatches allowed. Pairs in which this was not the case were discarded. As the final step, chimeric sequences were removed from the joined reads using the remove Bimera function of the DADA2 R package^40^. Subsequent taxonomic assignment was conducted by the uclust-consensus method from the QIIME^41^ microbial analysis framework using the Silva v. 123 reference database^42^. Samples with number of reads <1000 were removed from the analysis.

### Statistical methods

Four different matrices (maternal oral swabs, neonatal oral swabs, neonatal meconium, and neonatal transitional stool) were independently evaluated.

Both maternal and neonatal oral swabs were divided into those collected ≤48 h and those collected >48 h after childbirth. This cut-off was chosen to correspond to the meconium/transitional stool classification as meconium should pass ideally within the first 48 h in healthy full-term neonates^43^.

Contaminant ASVs were identified using the Decontam R package (v. 1.10.0)^44^. Neonatal samples were assumed to be low-abundant, hence the function IsNotContaminant with the default setting was used. For the mother’s oral swabs, the function IsContaminant with the default setting was used. Only the prevalence method was applicable. ASVs defined as contaminants were removed from all samples. Additionally, Cyanobacteria, mitochondria, and bacteria unassigned on the phylum level were filtered out. Finally, only genera with a relative abundance ≥0.5 % in at least one sample or with a relative abundance <0.5 % in at least three samples were included in the subsequent analysis.

All analyses were performed on genus, family, order, class, and phylum level. Prior to statistical analysis, data were treated as compositional and transformed using the centered log-ratio (CLR) transformation^45^ on the raw read abundance matrix. All zeroes in the original dataset were replaced by a constant of 0.65.

Fisher’s exact test (categorical variables) and Mann-Whitney U test (continuous variables) were used for the comparison of demographic and clinical characteristics among the groups of interest as well as for the comparison of alpha diversity indices.

Variability in the bacterial composition of different matrices was first visualized using multivariable approaches, namely Principal Component Analysis (PCA). The permutational multivariate analysis of variance (PERMANOVA; 9999 permutations) was performed to test for differences in the dispersion and centroids of the groups of bacterial communities, based on the Euclidean distance.

Bacterial co-occurrence was displayed by the UpSet plots. The presence of specific genera was considered as more than five reads in at least one sample in a defined material. Mann-Whitney U test was used to compare the differences in abundances of individual taxa between the groups. The comparison was performed only if the genus was present in at least three individuals in at least one of the compared groups. The resulting p-values were adjusted for multiple hypotheses testing using the Benjamini-Hochberg procedure (BH). Results were considered significant at FDR<0.1.

To evaluate the statistical power of this study, we conducted a simulation based on the Shannon index data across all four distinct sample types: maternal oral swabs, neonatal oral swabs, and neonatal meconium and transitional stool samples. A kernel density estimator was developed utilizing the measured Shannon index data, employing a Gaussian kernel with 2^14^ points, a bandwidth of 0.33, and an interval of [0, 6]. Subsequently, 1000 artificial datasets were generated for sample sizes ranging from 5 to 150 (in increments of 5) for both the +IAP and -IAP groups. For each artificial dataset, the Mann-Whitney U test was performed to determine statistical significance. The proportion of tests that yielded significance at the 0.05 level was then calculated to assess the study’s power.

All statistical analyses were performed in R (v. 4.0.5)^46^ using additional R packages: compositions (v. 2.0-4) for CLR transformation^47^; vegan (v. 2.6-2) for PERMANOVA^48^; factoextra (v. 1.0.7.) for PCA^49^ ggplot2 (v. 3.3.6) for box and whiskers plots and barplots^50^; FDRestimation (v. 1.0.1) for Benjamini-Hochberg correction^51^; UpSetR (v. 1.4.0.) for UpSet plots^52^.

### Ethics approval and consent to participate

The CELSPAC: TNG study was approved by the Multicentre and Local Ethical Committee of University Hospital Brno, Czech Republic (No. 20140409-01, date 09/04/2014) and performed according to relevant ethical regulations. Mothers gave informed consent for themselves and prospective neonates.

## Results

### Participant’s characteristics

Out of 100 pregnant women originally recruited from the CELSPAC: TNG, 66 mother-neonate pairs and their 198 samples met all inclusion and exclusion criteria and were included in this preliminary study (see the flowchart in Fig. 1), i.e., the drop-out was 34%. Of these, N=33 neonates were delivered by CS or vaginally with IAP (cases; 18 +IAP CS and 15 +IAP VD, respectively) and N=33 neonates were delivered vaginally with no IAP (controls, -IAP VD). We have also acquired data on the feeding mode from the hospital and found out that the number of exclusively breastfed, partially breastfed and formula-fed infants did not differ between the IAP groups during the hospital stay, with a clear majority (72%, 48 out of 66 neonates) exclusively breastfed (see Table 1).

**Table 1:** Demographic characteristics of the 66 mothers and neonates and characteristics of their samples.

Characteristics of mothers	+IAP	-IAP	p-value	Statistical test	+IAP CS	+IAP VD	-IAP VD	p-value	Statistical test
N	33	33			18	15	33		
Maternal age (in years)—median (min, max)	32 (19, 38)	33 (21, 43)	0.344	Mann-Whitney	30 (19, 37)	32 (28, 38)	33 (21, 43)	0.269	Kruskal-Wallis ANOVA
Gravidity (number of pregnancies in life) (%)									
I	12 (36.4)	11 (33.3)	1.000	Fisher‘s exact	9 (50.0)	3 (20.0)	11 (33.3)	0.475	Fisher‘s exact
II	13 (39.4)	13 (39.4)			5 (27.8)	8 (53.3)	13 (39.4)		
III and more	8 (24.2)	9 (27.3)			4 (22.2)	4 (26.7)	9 (27.3)		
Parity (number of parturitions) (%)									
I	16 (48.5)	13 (39.4)	0.376	Fisher‘s exact	12 (66.7)	4 (26.7)	13 (39.4)	0.092	Fisher‘s exact
II	15 (45.5)	14 (42.4)			6 (33.3)	9 (60.0)	14 (42.4)		
III	2 (6.1)	6 (18.2)			0 (0.0)	2 (13.3)	6 (18.2)		
Induced delivery (%)	13 (39.4)	7 (21.2)	0.180	Fisher‘s exact	8 (44.4)	5 (33.3)	7 (21.2)	0.204	Fisher‘s exact
Rupture of the amniotic sac (%)	5 (15.2)	2 (6.1)	0.427	Fisher‘s exact	2 (11.1)	3 (20.0)	2 (6.1)	0.384	Fisher‘s exact
Asthma (%)	2 (6.1)	3 (9.1)	1.000	Fisher‘s exact	1 (5.6)	1 (6.7)	3 (9.1)	1.000	Fisher‘s exact
Gestational diabetes (%)	2 (6.1)	3 (9.1)	1.000	Fisher‘s exact	1 (5.6)	1 (6.7)	3 (9.1)	1.000	Fisher‘s exact
Meconium in amniotic fluid (%)	4 (12.1)	3 (9.1)	1.000	Fisher‘s exact	1 (5.6)	3 (20.0)	3 (9.1)	0.441	Fisher‘s exact
Group B *Streptococcus* positive* (%)	7 (21.2)	0 (0.0)	0.011	Fisher‘s exact	2 (11.1)	5 (33.3)	0 (0.0)	0.001	Fisher‘s exact
Oral swab collection (%)									
Within 48 h after birth of her child	16 (48.5)	25 (75.8)	0.041	Fisher‘s exact	4 (22.2)	12 (80.0)	25 (75.8)	<0.001	Fisher‘s exact
After 48 h after birth of her child	17 (51.5)	8 (24.2)			14 (77.8)	3 (20.0)	8 (24.2)		

Characteristics of neonates	+IAP	-IAP	p-value	Statistical test	+IAP CS	+IAP VD	-IAP VD	p-value	Statistical test
N	33	33			18	15	33		
Gestational age (weeks) (%)									
38–39	17 (51.5)	8 (24.2)	0.041	Fisher‘s exact	9 (50.0)	8 (53.3)	8 (24.2)	0.076	Fisher‘s exact
40–41	16 (48.5)	25 (75.8)			9 (50.0)	7 (46.7)	25 (75.8)		
Sex (%)									
Female	15 (45.5)	17 (51.5)	0.806	Fisher‘s exact	9 (50.0)	6 (40.0)	17 (51.5)	0.766	Fisher‘s exact
Male	18 (54.5)	16 (48.5)			9 (50.0)	9 (60.0)	16 (48.5)		
Birth weight (g) median (min, max)	3600 (2350, 4340)	3390 (2630, 4630)	0.366	Mann-Whitney	3635 (2680, 4340)	3500 (2350, 4050)	3390 (2630, 4630)	0.298	Kruskal-Wallis ANOVA
Apgar score (%)									
Low (<7)	2 (6.1)	1 (3.0)	1.000	Fisher‘s exact	2 (11.1)	0 (0.0)	1 (3.0)	0.305	Fisher‘s exact
Normal (>7)	31 (93.9)	32 (97.0)			16 (88.9)	15 (100.0)	32 (97.0)		
Basal excess (Astrup) median (min, max)	−4.9 (−11.9, −0.7)	−5.3 (−13.6, −0.9)	0.119	Mann-Whitney	−4.9 (−11.9, −0.7)	−5.0 (−8.1, −1.6)	−5.3 (−13.6, −0.9)	0.257	Kruskal-Wallis ANOVA
pH median (min, max)	7.29 (7.07, 7.42)	7.31 (7.00, 7.47)	0.714	Mann-Whitney	7.29 (7.07, 7.36)	7.33 (7.19, 7.42)	7.31 (7.00, 7.47)	0.377	Kruskal-Wallis ANOVA
Newborn immunization (%)	4 (12.1)	1 (3.0)	0.355	Fisher‘s exact	3 (16.7)	1 (6.7)	1 (3.0)	0.172	Fisher‘s exact
Newborn infection (%)	1 (3.0)	3 (9.1)	0.613	Fisher‘s exact	1 (5.6)	0 (0.0)	3 (9.1)	0.802	Fisher‘s exact
Newborn conjunctivitis (%)	3 (9.1)	6 (18.2)	0.475	Fisher‘s exact	3 (16.7)	0 (0.0)	6 (18.2)	0.227	Fisher‘s exact
Newborn jaundice (%)	4 (12.1)	5 (15.2)	1.000	Fisher‘s exact	1 (5.6)	3 (20.0)	5 (15.2)	0.467	Fisher‘s exact
Feeding mode									
Exclusively breastfed	24 (72.7)	24 (72.7)	0.564	Fisher’s exact	14 (77.8)	10 (66.7)	24 (72.7)	0.644	Fisher’s exact
Partially breastfed	2 (6.1)	1 (3.0)			1 (7.1)	1 (6.7)	1 (3.0)		
Formula fed	6 (18.2)	4 (12.1)			2 (14.3)	4 (26.7)	4 (12.1)		
Missing	1 (3.0)	4 (12.1)			1 (7.1)	0	4 (12.1)		
Oral swab collection (%)									
Within 48 h after birth	16 (48.5)	27 (81.8)	0.009	Fisher‘s exact	4 (22.2)	12 (80.0)	27 (81.8)	<0.001	Fisher‘s exact
After 48 h after birth	17 (51.5)	6 (18.2)			14 (77.8)	3 (20.0)	6 (18.2)		
Fecal samples (%)									
Meconium	19 (57.6)	16 (48.5)	0.622	Fisher‘s exact	9 (50.0)	10 (66.7)	16 (48.5)	0.554	Fisher‘s exact
Transitional stool	14 (42.4)	17 (51.5)			9 (50.0)	5 (33.3)	17 (51.5)		

Data is presented as counts or median (percentage or minimum and maximum in brackets).

^*^Group B *Streptococcus* positive status includes those with positive screening status.

*N* number of cases, *+IAP* with intrapartum antibiotic prophylaxis, *-IAP* without intrapartum antibiotic prophylaxis, *CS* C-section, *VD* vaginal delivery, *N* number of participants.

The average maternal age was 32 ± 4 years. Even though mothers with gestational diabetes and asthma were enrolled in the study, these variables were represented equally among groups. Overall, there were no significant differences (p>0.05) in the mother’s or neonate’s health and birth characteristics between the +IAP and -IAP groups. There were significant differences in the numbers of collected oral swabs, from both mothers and neonates (p<0.05, p<0.01, respectively), grouped according to the sampling period; in mothers and neonates without IAP, oral swabs were collected predominantly ≤48 h after birth (75.8% and 81.8%, respectively) while oral swabs were collected >48 h after birth in 51.5% of both mothers and neonates with IAP. Neonatal birth weight ranged from 2350 to 4630 grams.

Administered antibiotics varied among the delivery modes. In women with CS delivery, cephalosporins (N=14), penicillins (N=3) or lincosamides (N=1, specifically clindamycin) were used. In the VD group, penicillins (N=14) were by far the most common antibiotics; cephalosporin was used only in one case with a premature rupture of membranes.

### Bacteriome analysis—general results

In total, 206 samples were analyzed, of which 198 met the quality criteria for further statistical analysis. After quality filtering and chimeras removal, 12,229,051 reads (median=56,823 reads per sample; interquartile range, IQR=25,835) were obtained, of which 4,045,330 reads (median=55,217.50 reads per sample; IQR=23,999.75) originated from the maternal oral swabs (N=66); 3,821,525 reads (median= 55,157 reads per sample; IQR=27,384.25) from the neonatal oral swabs (N=66); 2,255,835 reads (median=59,802 reads per sample; IQR= 28,281) from the meconium (N=35); and 2,106,361 reads (median=63,584 reads per sample; IQR= 34,087) from the transitional stools (N=31). After the decontamination step and filtering out ASV unassigned at the phylum level as well as Cyanobacteria*-* and mitochondria-assigned ASVs, most phyla (8 out of 12, i.e., 67%) were present at low abundances (median of relative abundance <5%).

**Figure 2:**
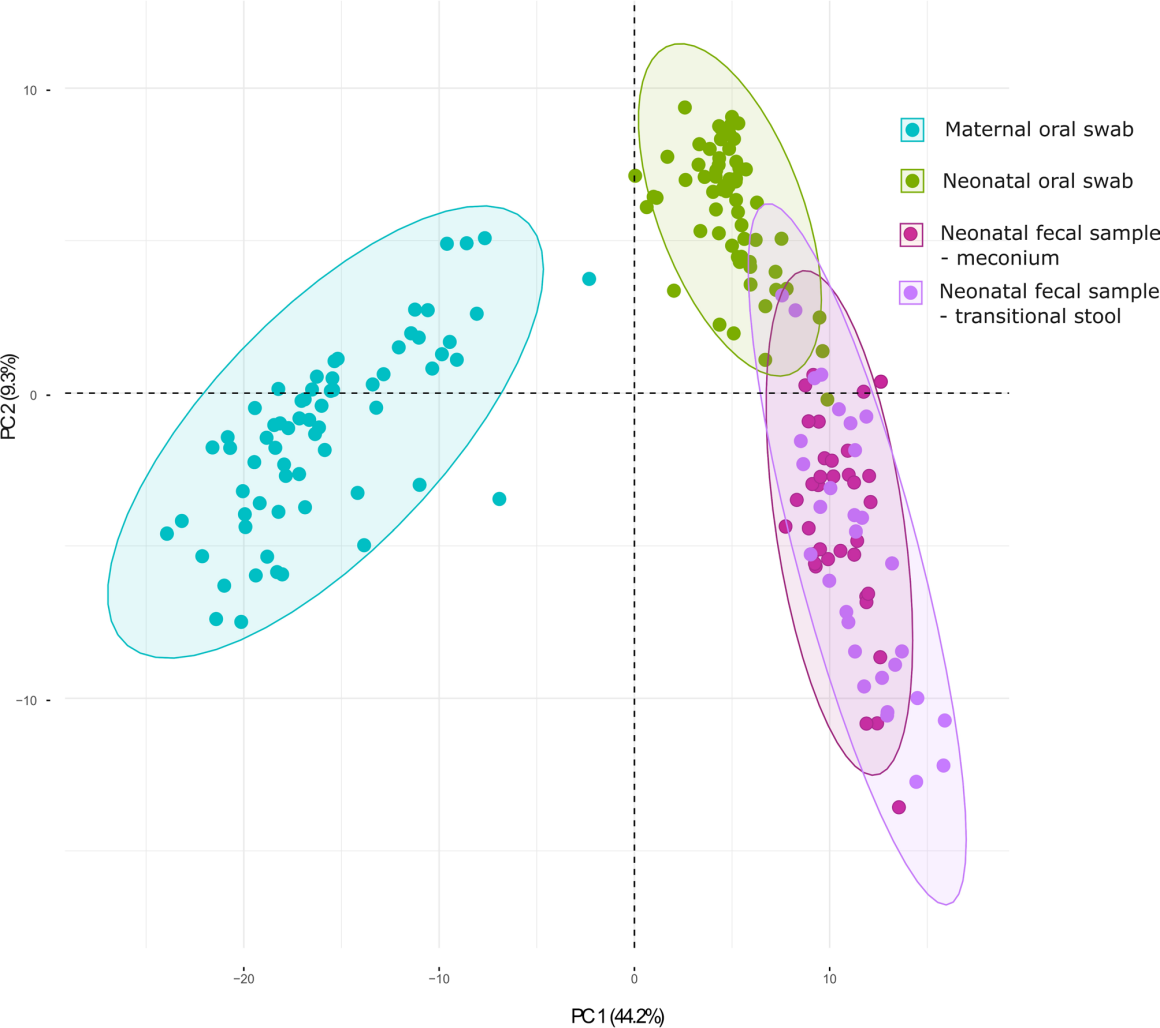
Principal component analysis (PCA, genus level) of maternal oral bacteriomes, neonatal oral and fecal bacteriomes.

**Figure 3:**
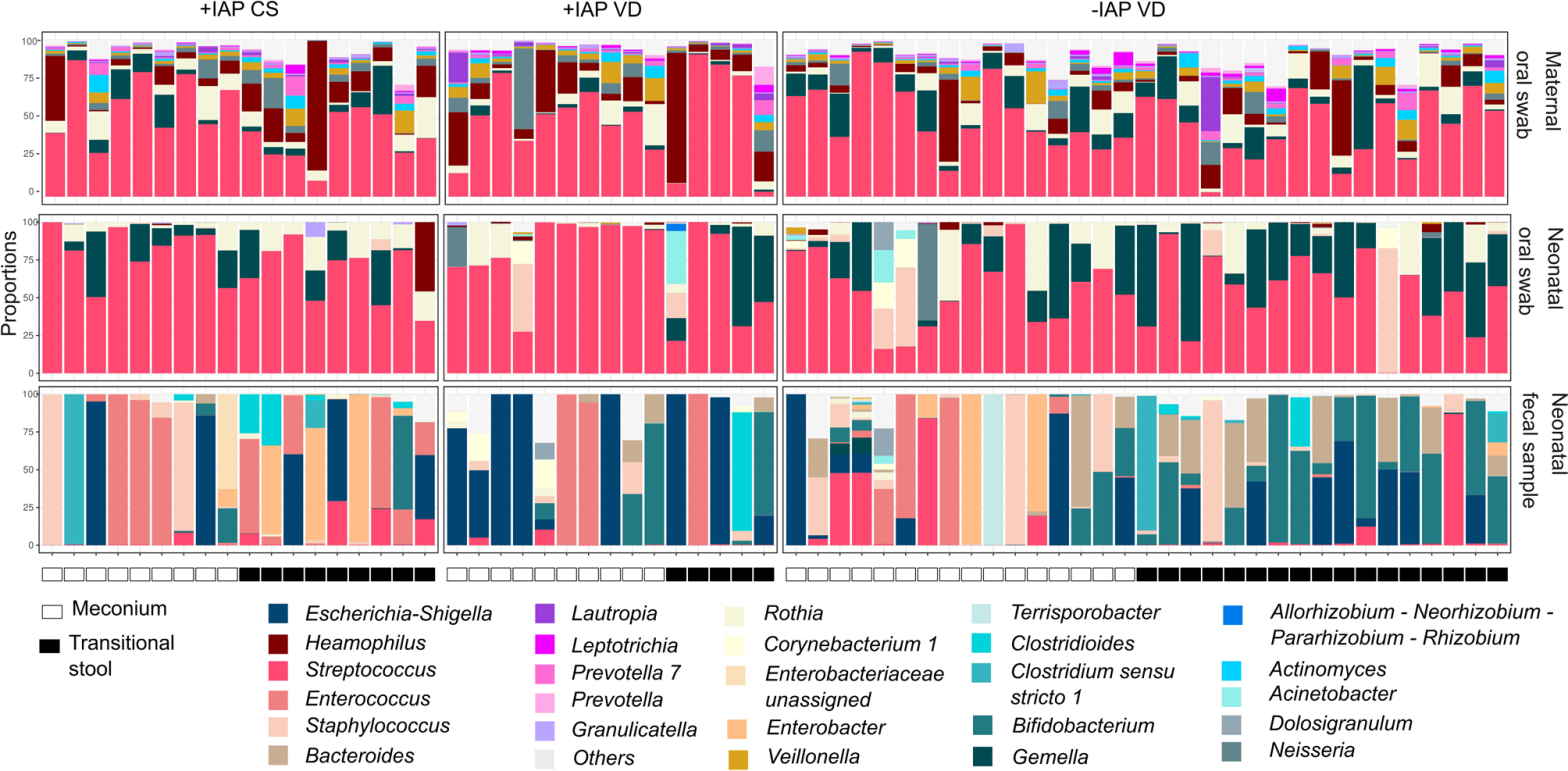
Bacteriome composition (genus level) in maternal oral swabs, neonatal oral swabs, and fecal samples. Samples classified according to intrapartum antibiotic exposure *+IAP* with intrapartum antibiotic prophylaxis, *-IAP* without intrapartum antibiotic prophylaxis, *CS* C-section, *VD* vaginal delivery.

Principal Component Analysis (PCA, Fig. 2) revealed the presence of three distinct bacterial communities representing the maternal oral, neonatal oral, and neonatal fecal bacteriomes. The bacteriome composition of the most abundant bacterial genera in maternal oral swabs, neonatal oral swabs, and fecal samples is shown in Fig. 3. Firmicutes, Proteobacteria*,* and Actinobacteria were the three most abundant phyla, with median relative abundances of 63.1, 12.5, and 9.2%, respectively. The numbers of genera were as follows: mothers’ oral swabs: 114 genera; neonates’ oral swabs: 28 genera; neonates’ meconium: 35 genera; neonates´ transitional stool: 25 genera.

Initial analyses of the samples revealed no statistically significant differences in the number of ASVs or Shannon index between the +IAP CS and +IAP VD groups in any of the neonatal matrices (see Table S1). For this reason, both these groups whom IAP was administered were combined into one for this type of analyses.

**Figure 4:**
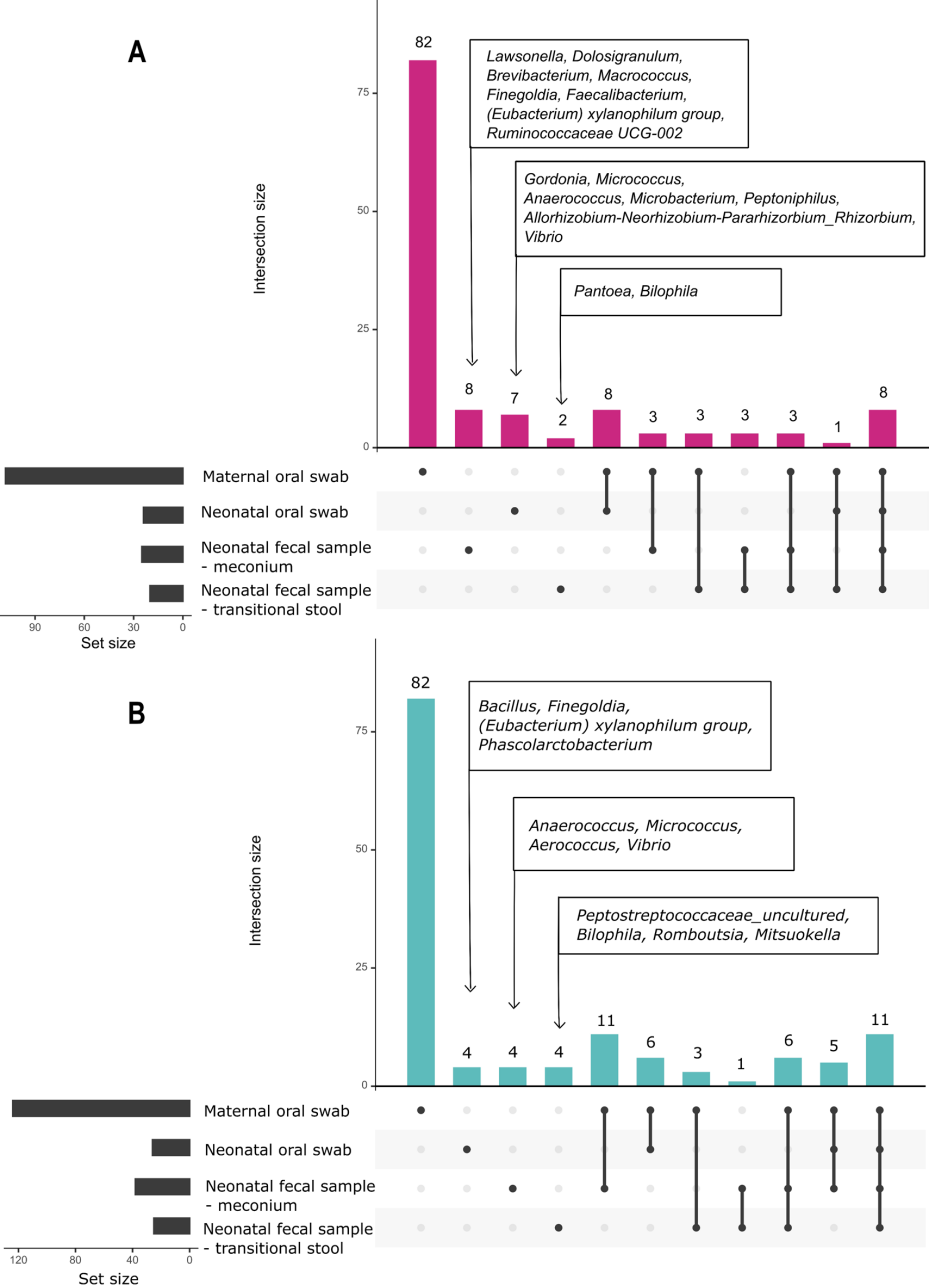
Upset plot of bacterial genera in maternal and neonatal samples. In the figure, dots signify the number of unique bacterial genera specific to each sample matrix. Connecting lines between dots indicate the number of genera shared among different sample matrices. (**A**) with administration of intrapartum antibiotic prophylaxis (+IAP) and (**B**) without administration of intrapartum antibiotic prophylaxis (-IAP).

To gain further insights into the bacterial composition overlap among these populations, an UpSet plot (Fig. 4) was constructed. An intersection between maternal and neonatal oral swabs was observed in 17 genera in the +IAP group and 22 genera in the -IAP group. In addition, *Haemophilus* and *Neisseria* were also among the most commonly observed genera in both maternal and neonatal oral samples. Furthermore, the overlap between maternal oral swabs and neonatal fecal samples revealed 18 genera present in the +IAP group and 36 genera in the -IAP group. *Bifidobacterium* and *Bacteroides* were among the dominant genera in this shared bacterial community.

In the maternal oral swabs, total of 82 unique bacterial genera were coincidentally observed both in +IAP and -IAP groups. Some examples of genera unique to maternal oral swabs to be named were *Prevotella*, *Corynebacterium*, and *Fusobacterium*. Lastly, there were bacterial genera that were not found in maternal oral swabs but were present in neonatal oral swabs (7 genera in +IAP and 4 genera in -IAP) and neonatal fecal samples (10 genera in +IAP and 8 in -IAP).

### Effect of IAP on the maternal oral bacteriome

The comparison of maternal oral bacteriomes after giving birth with or without IAP did not reveal any significant differences in alpha diversity of oral bacterial representatives (p>0.05, Fig. S1). In addition to dominating genera *Streptococcus*, *Rothia*, and *Gemella* (Fig. 3)*,* common oral genera such as *Prevotella, Veillonella, Fusobacterium,* and *Actinomyces* were found in maternal oral swabs as well. The PCA showed differences between +IAP and -IAP maternal oral samples; however, the PERMANOVA test was not significant (p=0.056).

### Effect of IAP on the neonatal oral bacteriome

The effect of IAP on the neonatal bacteriome was tested separately for oral samples collected ≤48 h and >48 h after birth. IAP was associated with significantly reduced bacteriome diversity in neonatal oral samples (Shannon index; p=0.01) ≤48 h after birth (Fig. S2). In samples collected >48 h after birth, an insignificant decrease (p>0.05) in both the number of amplicon sequence variants (ASVs) and the Shannon index was observed in oral neonatal samples in the +IAP group compared to the control (-IAP) group. The PERMANOVA test revealed a significant impact of IAP on neonatal oral bacteriomes ≤48 h after birth (p=0.038) but no difference was observed in samples collected >48 h after birth (p>0.05).

**Figure 5:**
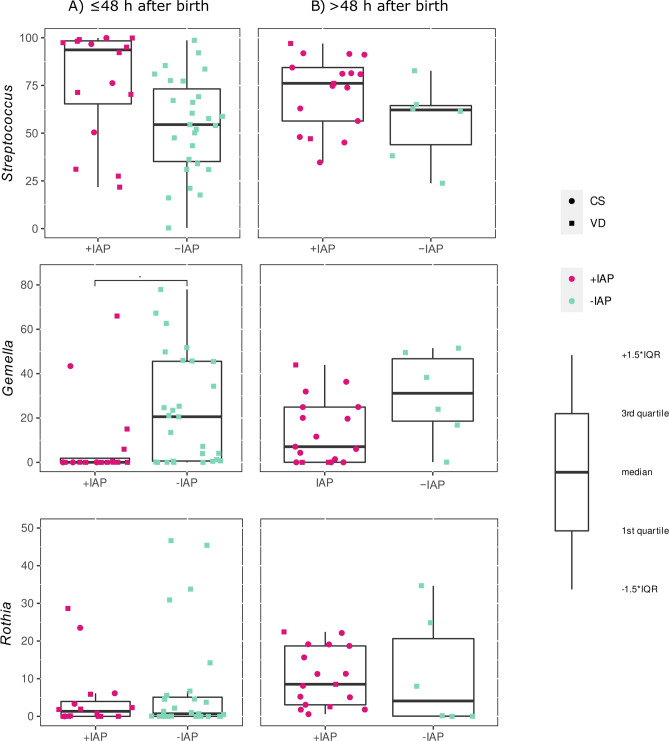
Relative abundance of specific bacteria in neonatal oral swabs. This figure examines the relative abundance of *Streptococcus*, *Gemella*, and *Rothia* in neonatal oral swabs according to the intrapartum antibiotic prophylaxis: (**A**) samples collected ≤48 h after birth, (**B**) samples collected >48 h after birth; +IAP, with intrapartum antibiotic prophylaxis; *-IAP* without intrapartum antibiotic prophylaxis, *IQR* interquartile range, *CS,*C-section, *VD* vaginal delivery. *q<0.1.

Bacterial genera *Streptococcus, Gemella*, and *Rothia* dominated in neonatal oral bacteriomes in both groups*.* In the +IAP group, the relative abundance of the genus *Gemella* was significantly lower in neonatal oral samples collected ≤48 h after birth (q=0.08) than in the controls (-IAP). This was, however, not true of samples collected >48 h after birth (p>0.05, q>0.1, Fig. 5). Significant differences in relative abundances between cases and controls were also observed on the family level in Neisseriaceae (q=0.09) and Streptococcaceae (q=0.09), data are not shown.

### Effect of IAP on the neonatal fecal bacteriomes

When all neonate fecal (both meconium and transitional stool) samples (N=66) were stratified according to the antibiotic exposure during delivery, a statistically significant difference in the number of ASVs (p<0.001) between +IAP and -IAP group was observed.

However, meconium samples alone did not show any significant difference in the number of observed ASVs (p>0.05) or in the Shannon index (p>0.05) between the tested groups. On the other hand, transitional stool samples differed significantly in the number of observed ASVs in +IAP neonates compared to the -IAP neonates (p=0.02, Fig. S3).

**Figure 6:**
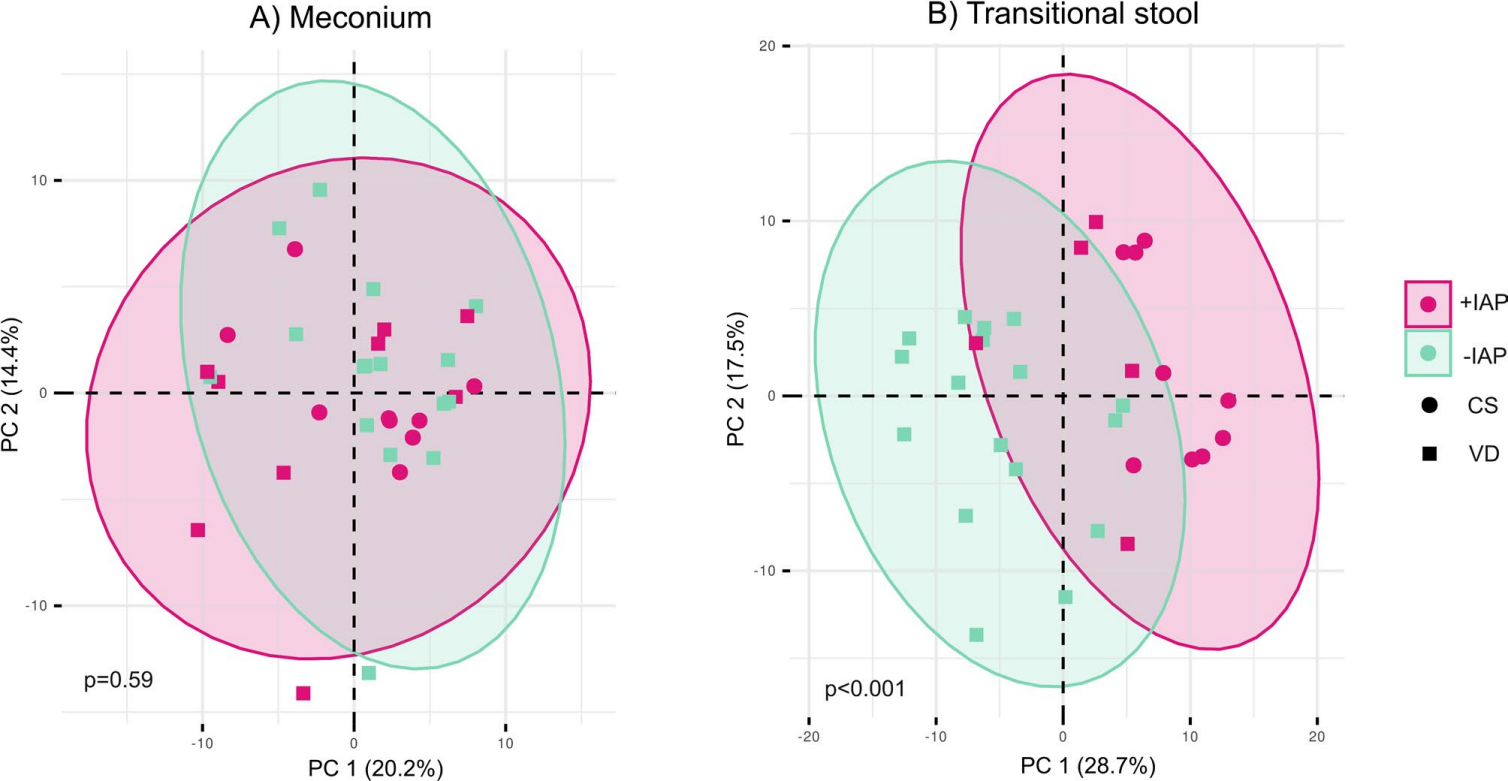
Principal component analysis (PCA) of neonatal meconium and transitional stool samples. Displaying a PCA (genus level) of neonatal (**A**) meconium and (**B**) transitional stool samples according to the intrapartum antibiotic prophylaxis; *+IAP* with intrapartum antibiotic prophylaxis, *-IAP* without intrapartum antibiotic prophylaxis, *CS* C-section, *VD* vaginal delivery.

The PERMANOVA test on the bacterial profiles assessing the meconium and transitional stool separately revealed significant differences in neonatal transitional stool bacteriome at the genus level between the two studied groups (p<0.001, Fig. 6), while no significant differences were observed in meconium samples (p=0.59).

**Figure 7:**
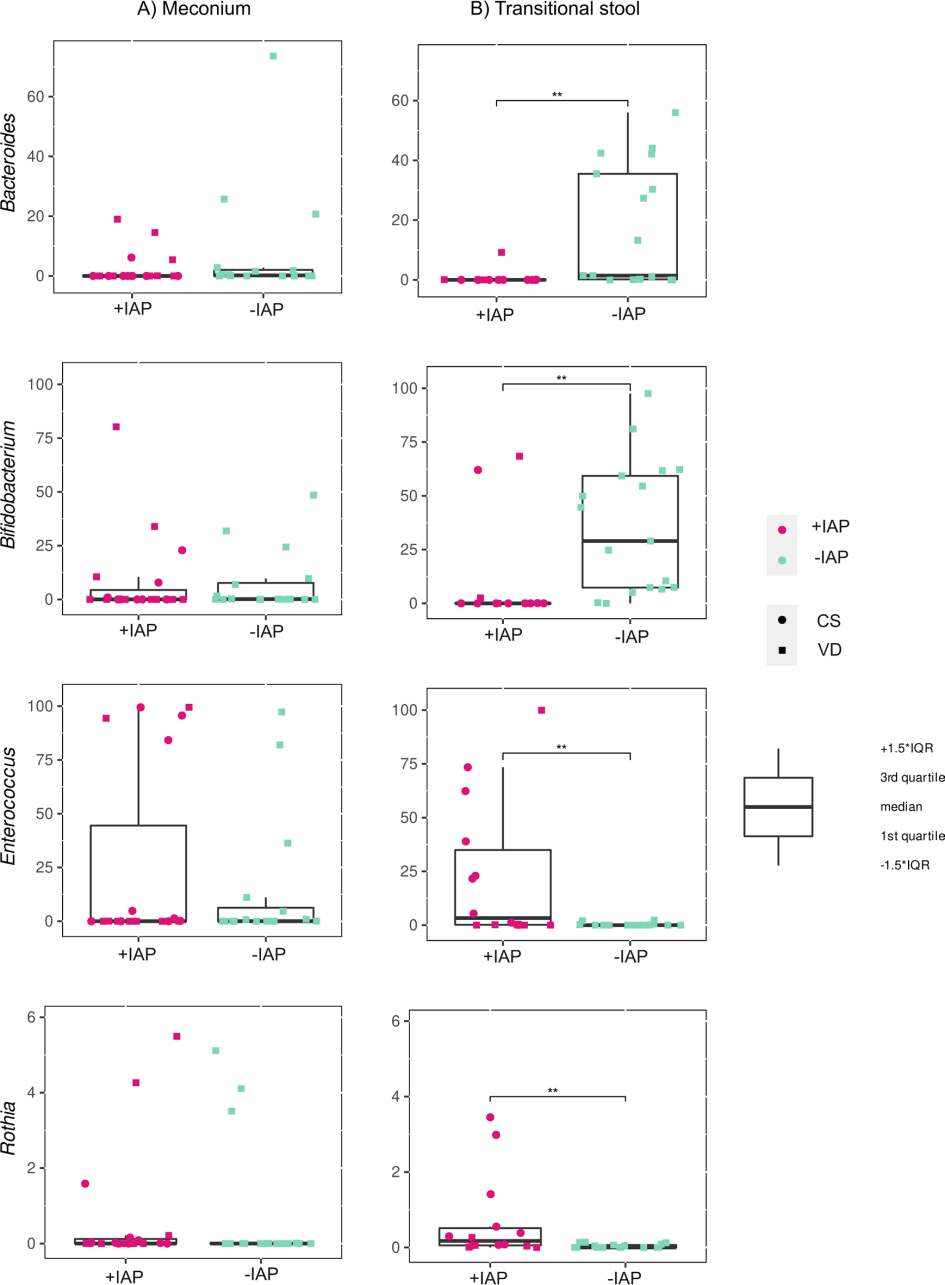
Relative abundance of specific bacteria in stool samples: This figure illustrates the relative abundance of *Bacteroides, Bifidobacterium, Enterococcus,* and *Rothia* in neonatal (**A**) meconium and (**B**) transitional stool according to the intrapartum antibiotic prophylaxis; *+IAP* with intrapartum antibiotic prophylaxis, *-IAP* without intrapartum antibiotic prophylaxis, *IQR* interquartile range, *CS* C-section, *VD* vaginal delivery; **q<0.01.

IAP administration affected the abundance of several bacteria in transitional stool samples, but not in meconium samples. The taxonomical analysis identified the five most abundant genera in meconium samples as *Staphylococcus, Escherichia-Shigella, Streptococcus, Bifidobacterium*, and *Enterococcus* (Fig. 3). *Bifidobacterium, Streptococcus, Escherichia-Shigella, Staphylococcus*, and *Enterococcus* were the most abundant genera in transitional stool samples. IAP was significantly associated with decreased relative abundances of the genera *Bifidobacterium* (q=0.002) *Bacteroides* (q=0.003) and *Parabacteroides* (q=0.07) and significantly higher relative abundances of *Rothia* (q=0.005), *Enterococcus* (q=0.01, see Fig. 7), and *Clostridioides* (q=0.09) in transitional stool samples (Mann-Whitney U test).

In addition, we have separately analyzed the representation of individual bacterial genera among all three groups (-IAP VD, +IAP VD and +IAP CS). This analysis revealed that in transitional stool samples (but not meconium samples), the relative abundance of the genera *Rothia, Enterococcus, Bifidobacterium,* and *Bacteroides* significantly differed among the groups (Kruskal-Wallis test, q<0.05). This difference was clearly driven by the increased relative abundance of *Rothia* and *Enterococcus* in +IAP CS neonates compared to the -IAP VD group (Dunn’s post hoc test, q<0.05) as well as by the higher relative abundances of the genera *Bifidobacterium* and *Bacteroides* in the -IAP VD group than in the +IAP CS group (q<0.05). Neither IAP nor mode of delivery affected the relative abundance of *Lactobacillus* or *Clostridioides* in the meconium and transitional stool samples*.*

## Discussion

In this article, our primary objective was to investigate the impact of IAP on neonatal gastrointestinal bacteriomes, with a particular focus on the first week of life. We sought to elucidate this relationship by characterizing the maternal and neonatal oral bacteriomes and the bacteriomes of neonatal meconium and transitional stool. This approach allowed us to understand how IAP might shape early neonatal microbiota development and, therefore, improve our understanding of the foundational days of the human gastrointestinal microbiome.

Maternal oral swabs displayed a higher diversity in bacterial composition than neonatal oral swabs, which is consistent with previous research that has suggested the adult oral microbiota is typically more diverse compared to the neonatal one^53,54^. As expected, no effect of IAP on the maternal oral bacteriome was found in our study. This aligns with the concept that established microbial communities, such as that of the adult oral cavity, are more resilient to disturbances, including antibiotic interventions^55,56^. However, the antibiotic exposure during delivery affected the diversity of neonatal oral and transitional stool bacteriomes in the first week of life, reflecting the susceptibility of these developing bacterial communities to external interventions.

### Impact of IAP on the neonatal oral bacteriome in the first week of life

The oral cavity presents an initial entry point to microbial colonization of gastrointestinal tract. Nevertheless, there is a limited understanding to the effect of IAP on neonatal oral microbiota development in the first week after birth. Our study showed significant changes in bacterial diversity and abundance in the first 48 h after IAP exposure. However, our data suggests that ≤48 h after birth, the impact of IAP on neonatal oral bacteriome is suppressed.

In the first 48 h after birth, IAP exposure affected the abundances of the genus *Gemella* and families Neisseriaceae and Streptococcaceae in the neonatal oral cavity. This is in agreement with the study by Gomez-Arango et al.^26^ who investigated the effect of IAP on neonatal oral bacteriome within the first 3 days after birth by CS in 36 neonates. Their results pointed to a decreased relative abundance of Streptococcaceae, Gemellaceae, and Lactobacillales, and an increased abundance of Neiseeriaceae or Prevotellaceae in the oral cavity of neonates exposed to IAP^26^. The genera *Streptococcus, Staphylococcus, Veillonella*, and *Lactobacillus* are recognized as the first oral colonizers^55,57,58^. *Streptococcus* is one of the most dominant genera found in the oral microbiome of neonates during their first days of life^59–61^. This is due to several factors, including the fact that *Streptococcus* species are naturally present in the maternal vaginal microbiota, which is one of the first microbial communities that the neonates are exposed to during VD^10^. Additionally, *Streptococcus* species are well-adapted to the oral cavity environment^62,63^. The genus *Gemella,* together with *Granulicatella, Haemophilus*, and *Rothia,* count among later colonizers, the abundance of which grows with the increasing age of the infant^57,58,62^. The exact role of *Gemella* and *Neisseria* in the oral cavity of neonates is not yet fully understood, but it is believed that they may play a role in the early colonization of the oral cavity and contribute to the development of a healthy microbial community^26,64^. *Neisseria* species are Gram-negative bacteria that are commonly found on the mucosa of the respiratory tract and oral cavity and may play also an important role in the early development of a healthy oral microbial community^61,65^.

Studies exploring the impact of IAP administered during CS have shown that IAP can have short-term effects on the oral microbiota of neonates^8,10,26,66–68^. An Irish birth cohort study with 84 neonates highlighted the differences in bacteriomes in the first week of the lives of neonates delivered by CS and VD; later, the fingerprint of CS vanished^67^. A Swedish birth cohort study with 59 neonates^68^ did not reveal any association between the delivery mode and neonatal oral bacteriome composition. Unfortunately, they did not investigate differences caused by application of IAP in VD neonates.

It is important to note that the oral microbiota is dynamic and unstable in the first days after birth and can change over time due to various factors. With time, the differences in oral microbiota associated with IAP seem to diminish as other factors come into play in shaping the oral microbiota in infants, such as diet and environmental influence.

### Impact of IAP on the neonatal fecal bacteriome in the first week of life

Our study demonstrates that IAP can influence the microbiota of transitional stool but not that of the meconium. Specifically, IAP was associated with decreased relative abundances of the genera *Bifidobacterium* and *Bacteroides* in the transitional stool samples in both CS and VD neonates exposed to IAP compared to VD neonates without IAP exposure. Our results correspond to the evidence of the IAP effect on gut bacterial community 3–7 days after birth^69^. One of the first culture-based bacteriological studies, performed by Jauréguy et al.^70^ in 2004, examined amoxicillin used in GBS prophylaxis and its effect on neonates 3 days after birth, predominantly in neonates born vaginally. Their results showed that colonization by *Bifidobacteria* was detected in fewer No-IAP neonates than in IAP neonates. The topic was followed by more than 20 culture-independent bacteriological studies investigating IAP and gut microbiota development since 2015^71^. Only some of the studies evaluated the effect of IAP in the first days after birth in full-term neonates^12,21,27,30,31,69,70,72–74^. Stearns et al.^21^, and Dierikx et al.^75^ presented delayed colonization by Actinobacteria in both CS and VD with IAP neonates on the postpartum Days 3 and 7. Stearns et al.^21^ observed a decrease in *Bifidobacterium* and *Bacteroides* spp. followed by a significant increase in Lachnospiraceae in CS neonates compared to VD neonates unexposed to IAP. Bacteriomes of neonates with IAP did not differ between those born through CS and VD. Furthermore, Dierikx et al.^75^ found no differences in the bacteriome on the phylum level between vaginally and CS-born neonates on Days 1 and 7. However, on the genus level, they point to a decreased abundance of *Bacteroides* with a concurrent increase in *Enterococcus* in CS neonates compared to VD neonates on Day 7.

Studies focusing entirely on VD neonates of mothers positive for GBS screening present similar findings. Mazzola et al.^76^ found differences within the Enterobacteriaceae family and within the *Bifidobacterium* and *Bacteroides* genera in stool samples 7 days after birth. By qPCR quantification of selected bacteria, Aloisio et al.^30^ and Corvaglia et al.^74^ also showed a significant average reduction in the counts of *Bifidobacterium* spp. in neonates whose mothers received IAP. Nogacka et al.^12^ observed a reduction in the levels of Actinobacteria and Bacteroidetes and an increase of Proteobacteria and Firmicutes in IAP neonates in comparison to those without IAP. However, at 10 days of age, these differences reached statistical significance only for Actinobacteria. Last but not least, Tapianinen et al.^27^ observed a significant difference between the IAP and control groups in the relative abundance of *Bacteroides* on the third day*,* but no significant differences in any species in the first day after birth.

Furthermore, our study also revealed an increased relative abundance of the genus *Rothia* in CS neonates compared to VD neonates. In addition, the relative abundance of *Enterococcus* was significantly increased in transitional stool samples in +IAP CS neonates compared to -IAP VD neonates and borderline significantly increased when comparing +IAP CS to the +IAP VD neonates. Whether any of these changes are associated with the general fact that IAP was used, with specific antibiotics administered during the labor, or with the route of exposure to the microorganisms during the delivery needs to be further investigated. Future studies should ideally stratify the number of neonates according to the mode of delivery and IAP.

In line with our expectations, IAP treatment did not affect bacterial abundance or diversity in meconium samples in our study. More frequent meconium/transitional stool sampling in the first hours and days after birth could further help in explaining the early impact of the delivery mode and IAP on neonatal gut microbiota development. Meconium samples from the first two days after birth were investigated in a few other studies^27,31,73,75^. Similar to our findings, none of the previous studies found significant differences in any of the abovementioned bacterial groups in IAP-exposed neonates. The impact of IAP is, however, commonly reflected in gut microbiota later than two days after the birth.

### Strengths and limitations of the study

This is the first study examining the effect of IAP on neonatal bacteriome of the oral cavity, meconium, as well as transitional stool in the first week after birth. The accumulated evidence from previous metagenomic studies has shown inconsistent findings that may be attributed to factors such as varying antibiotic regimens, routes of administration, statistical analytical methods employed, or uncontrolled factors. Even though meconium and transitional stool samples are characterized by low microbial load, the use of an internal standard in our study offsets the analytical challenges. Furthermore, in this study, several negative controls were implemented to detect bacterial contaminants. Moreover, the quality of the data from marker-gene sequencing was further improved by removing contaminant DNA sequences by Decontam^45^.

In our study, we strictly adhered to rigorous inclusion and exclusion criteria to ensure the reliability and validity of our findings. Out of the 100 pregnant women originally recruited, only 66 mother-neonate pairs and their 198 samples met all the specified criteria, leading to a drop-out rate of 34%. One of the primary challenges we encountered was the logistical difficulty in collecting all required samples, particularly from mothers in the Intensive Care Unit after C-section. Another common cause of exclusion was the absence of crucial data (type of antibiotics provided) or samples. This selection process was necessary to control for various confounding factors and ensure a homogeneous study population of overall healthy mothers with no significant differences (p>0.05) in the mother’s or neonate’s health and birth characteristics between the +IAP and -IAP groups. Even though mothers with asthma and gestational diabetes were included in the study, they were equally distributed in both groups. It might be also objected that the difference in antibiotic treatment between the groups is also a limitation of the study. However, as cephalosporins and penicillins that were used in a vast majority of mothers in both groups have similar modes of action and, in effect, similar antimicrobial profiles, this limitation should not pose a problem for the validity of our results.

Our study describes the period when lactation is initiated and breastfeeding (including the contact with the maternal breast or the additional contact with other materials) can affect the neonates’ microbiota. Nogacka et al. observed different responses of *Bacteroides* to IAP depending on the presence or absence of breastfeeding^12^. In our study, only ten neonates evenly distributed among study groups were formula-fed. The low number of formula-fed neonates in individual groups precluded the subanalysis of feeding mode in the relation to neonatal oral and fecal bacteriomes. However, the even distribution of feeding modes ensures that our results are not bias by this factor.

The relatively small sample size in both the -IAP and +IAP groups is another limitation of our analysis. To help future studies similar to the presented one, we have performed a power analysis based on our results to determine the sample size for neonatal oral swabs and transitional stool samples that is necessary for achieving 80% power to detect a statistically significant difference in the Shannon index in neonatal oral swabs (85 individuals in each group) and transitional stool samples (53 individuals in each group).

## Conclusions

The current preliminary study describes bacteriomes of maternal and neonatal oral swabs and neonatal meconium and transitional stool to characterize the relationship between IAP and neonatal microbiota development in the first week of their life. No significant effects of IAP on maternal oral bacteriome in the first week after delivery were found. Exposure to IAP influences the oral bacteriome of neonates within the first 48 h after birth. However, the effect of IAP seems to diminish later in the first week of their life. Where meconium and stool are concerned, the differences in bacterial abundances due to IAP exposure are not reflected in meconium samples but transitional stool samples are affected by IAP. These findings highlight how antibiotics influence neonatal early bacterial development and point to the need for more research to understand the impact on children’s health in the long term.

## Supplementary Information

Supplementary Figure S1.

Supplementary Figure S2.

Supplementary Figure S3.

Supplementary Table S1.

Supplementary Legends.

## Data Availability

The data set from 16S rRNA amplicon sequencing can be accessed at the National Center for Biotechnology Information (NCBI) under the accession number PRJNA1036118 (https://www.ncbi.nlm.nih.gov/search/all/?term=PRJNA1036118). Other data are available upon request from the corresponding author.

## Abbreviations

CS: Cesarean section
GBS: Group B *Streptococcus*
IAP: Intrapartum antibiotic prophylaxis
NGS: Next-generation sequencing
PCR: Polymerase chain reaction
PCA: Principal component analysis
VD: Vaginal delivery
8-MOP: 8-methoxypsoralen
