# Supplementary material for: Impact of intrapartum antibiotic prophylaxis on the oral and fecal bacteriomes of children in the first week of life
Source: Sci Rep. 2024 Aug 6;14:18163. doi: 10.1038/s41598-024-68953-z (PMC11303690; doi:10.1038/s41598-024-68953-z)
Supplement: Supplementary file 4 — Supplementary Table S1. [file 41598_2024_68953_MOESM4_ESM.pdf]

Supplementary Material

Table S1. Comparison of numbers of ASVs and Shannon indices in neonatal oral and fecal samples between + IAP groups

	Neonatal oral swab				
	n	Number of ASVs		Shannon index	
		median	*p*-value	median	*p*-value
+IAP CS	18	9	0.899	1.149	0.145
+IAP VD	15	9		0.510	

	Neonatal meconium				
	n	Number of ASVs		Shannon index	
		median	*p*-value	median	*p*-value
+IAP CS	9	10	0.388	0.747	0.065
+IAP VD	10	7		1.026	

	Neonatal transitional stool				
	n	Number of ASVs		Shannon index	
		median	*p*-value	median	*p*-value
+IAP CS	9	13	0.592	1.646	0.083
+IAP VD	5	9		0.373	

N, number of cases; ASVs, amplicon sequence variants; +IAP, with intrapartum antibiotic prophylaxis; -IAP, without intrapartum antibiotic prophylaxis; CS, C-section; VD, vaginal delivery
