# Supplementary material for: Impact of intrapartum antibiotic prophylaxis on the oral and fecal bacteriomes of children in the first week of life
Source: Sci Rep. 2024 Aug 6;14:18163. doi: 10.1038/s41598-024-68953-z (PMC11303690; doi:10.1038/s41598-024-68953-z)
Supplement: Supplementary file 5 — Supplementary Legends. [file 41598_2024_68953_MOESM5_ESM.docx]

**Supplementary Material**

**Figure S1. The effect of intrapartum antibiotic prophylaxis on alpha diversity in maternal oral bacteriomes:** This figure displays **A)** samples collected ≤48 h after giving birth, **B)** samples collected >48 h after giving birth; ASVs, amplicon sequence variants; +IAP, with intrapartum antibiotic prophylaxis; -IAP, without intrapartum antibiotic prophylaxis; CS, C-section; VD, vaginal delivery

**Figure S2. The effect of intrapartum antibiotic prophylaxis on alpha diversity in neonatal oral bacteriomes**. This figure displays **A)** samples collected ≤48 h after birth, **B)** samples collected >48 h after birth; ASVs, amplicon sequence variants; +IAP, with intrapartum antibiotic prophylaxis; -IAP, without intrapartum antibiotic prophylaxis; CS, C-section; VD, vaginal delivery. * p<0.05

**Figure S3.** **Bacterial alpha diversity of neonatal fecal samples.** The figure illustrates **A)** meconium and **B)** transitional stool samples according to the intrapartum antibiotic prophylaxis; ASVs, amplicon sequence variants; +IAP, with intrapartum antibiotic prophylaxis; -IAP, without intrapartum antibiotic prophylaxis; IQR, interquartile range; CS, C-section; VD, vaginal delivery; * p<0.05

**Table S1. Comparison of numbers of ASVs and Shannon indices in neonatal oral and fecal samples between + IAP groups**

N, number of cases; ASVs, amplicon sequence variants; +IAP, with intrapartum antibiotic prophylaxis; -IAP, without intrapartum antibiotic prophylaxis; IQR, interquartile range; CS, C-section; VD, vaginal delivery
